# Supplementary material for: O-methyltransferase-like enzyme catalyzed diazo installation in polyketide biosynthesis
Source: Nat Commun. 2023 Sep 4;14:5372. doi: 10.1038/s41467-023-41062-7 (PMC10477347; doi:10.1038/s41467-023-41062-7)
Supplement: Supplementary file 1 — Supplementary information file [file 41467_2023_41062_MOESM1_ESM.pdf]

Supplementary information

O-methyltransferase-like enzyme catalyzed diazo installation in polyketide biosynthesis

Yuchun Zhao[1#], Xiangyang Liu[1#], Zhihong Xiao[1], Jie Zhou[1], Xingyu Song[2], Xiaozheng Wang[1], Lijun Hu[3], Ying Wang[3], Peng Sun[4], Wenning Wang[2], Xinyi He[1], Shuangjun Lin[1], Zixin Deng[1], Lifeng Pan[5], Ming Jiang[1]*

[1]State Key Laboratory of Microbial Metabolism, Joint International Research Laboratory of Metabolic & Developmental Sciences, and School of Life Sciences and Biotechnology, Shanghai Jiao Tong University, Shanghai 200030, P. R. China.

[2]Ministry of Education Key Laboratory of Computational Physical Sciences, Department of Chemistry, Institutes of Biomedical Sciences, Fudan University, Shanghai 200438, China

[3]Guangdong Province Key Laboratory of Pharmacodynamic Constituents of TCM and New Drugs Research, Center for Bioactive Natural Molecules and Innovative Drugs Research, Jinan University, Guangzhou 510632, P. R. China.

[4]School of Pharmacy, Second Military Medical University, 325 Guo-He Road, Shanghai 200433, P. R. China.

[5]State Key Laboratory of Bioorganic and Natural Products Chemistry, Shanghai Institute of Organic Chemistry, University of Chinese Academy of Sciences, Chinese Academy of Sciences, Shanghai 200032, China.

[#]These authors contributed equally.

*Corresponding author. Email: jiangming9722@sjtu.edu.cn

Table of Contents

Supplementary Tables... 4

Supplementary Table 1. Strains and plasmids used in this study...................................... 4

Supplementary Table 2. Primers used in this study... 6

Supplementary Table 3. Protein sequence homology of AlpH homologues...................... 6

Supplementary Table 4. Methyltransferases used for phylogenetic analysis and sequence alignment... 8

Supplementary Table 5. Statistics of X-ray crystallographic data collection and model refinement. 9

Supplementary Table 6. ^{1}H NMR and ^{13}C NMR data for glutamylhydrazine in D_2O... 10

Supplementary Table 7. ^{1}H NMR data comparison between reported prekinamycin and our isolated prekinamycin. 11

Supplementary Table 8. ^{13}C NMR data comparison between reported prekinamycin and our purified prekinamycin.. 12

Supplementary Figures 12

Supplementary Figure 1. The organization of biosynthetic gene clusters........................ 13

Supplementary Figure 2. *In vivo* characterization of Alp1W... 14

Supplementary Figure 3. SDS-PAGE analysis of purified AlpJ, AlpK, and AlpH......... 15

Supplementary Figure 4. *In vitro* characterization of Alp1W.. 16

Supplementary Figure 5. LC-HRMS analysis (negative mode) of 1................................ 17

Supplementary Figure 6. Sequence alignment of AlpH and its homologous proteins.... 18

Supplementary Figure 7. Proposed biosynthetic pathway of kinamycin......................... 19

Supplementary Figure 8. HRMS analysis (positive mode) of 2... 20

Supplementary Figure 9. HRMS analysis (positive mode) of 3... 21

Supplementary Figure 10. UV absorbance spectrum and LC-HRMS analysis of prekinamycin (4).. 22

Supplementary Figure 11. HPLC and HRMS analysis of the decomposition products of 5. 23

Supplementary Figure 12. HPLC analysis of the reactions with different combinations of enzymes and cofactors for the one-pot reaction. 24

Supplementary Figure 13. LC-HRMS analysis (negative mode) of 6............................... 25

Supplementary Figure 14. Effects of exogenous SAM, SAH, and Sinefungin supplemented to AlpJKH-catalyzed reaction. 26

Supplementary Figure 15. LC-MS analysis of chemically denatured AlpH.................... 27

Supplementary Figure 16. HPLC profiles of the reactions catalyzed by wild-type AlpH and its mutants.. 28

Supplementary Figure 17. Phylogenetic tree of AlpH and its homologues with other natural product methyltransferases (NPMTs)... 29

Supplementary Figure 18. AlpH does not affect the production of compound 2. 30

Supplementary Figure 19. HPLC analysis of the competition reactions using different concentrations of NAC. 31

Supplementary Figure 20. Analysis of the effect of isoleucine. 32

Supplementary Figure 21. Structural comparison analyses of the overall structure of AlpH with the OMT LaPhzM and MmcR as well as the pericyclase PdxI 33

Supplementary Figure 22. Detailed structural comparison of AlpH and the SAM-dependent OMT MmcR in complex with SAH 35

Supplementary Figure 23. MD simulations of the ligand-bound systems. 37

Supplementary Figure 24. SDS-PAGE analysis of purified AlpH and its mutants......... 38

Supplementary Figure 25. Proposed mechanism of C-N and C-C bond formation catalyzed by AlpH.. 39

Supplementary Figure 26. Surface plasmon resonance measurement of binding affinity between AlpH and gluN_2H_3. 40

Supplementary Figure 27. ^{1}H NMR spectrum of glutamylhydrazine in D_2O. 41

Supplementary Figure 28. ^{13}C NMR spectrum of glutamylhydrazine in D_2O. 42

Supplementary Figure 29. ^{1}H NMR spectrum of prekinamycin in $CDCl_3$. 43

Supplementary Figure 30. ^{13}C NMR spectrum of prekinamycin in $CDCl_3$. 44

The original SDS-PAGE gels 45

Supplementary references 47

Supplementary Tables

Supplementary Table 1. Strains and plasmids used in this study.

Strain or plasmid	Relevant properties	Source or reference
**Strains**		
DH10B	General cloning and plasmid maintenance	GibcoBRL
ET12567/pUB307	Used for intergeneric conjugation	[1]
ET12567/pUZ8002	Used for intergeneric conjugation	[2]
BW25113/pIJ790	λ Red, *cat, araC, rep101*	[3]
BL21(DE3)/pGro7	BL21(DE3) containing plasmid pGro7	TAKARA
BL21/pGro7/ pET28a-*alpH*	Expression strain of AlpH	This study
BL21/pGro7/ pET28a-*alpH* G203A	Expression strain of AlpH G203A	This study
BL21/pGro7/ pET28a-*alpH* K266A	Expression strain of AlpH K266A	This study
BL21/pGro7/ pET28a-*alpH* K266D	Expression strain of AlpH K266D	This study
BL21/pGro7/ pET28a-*alpH* H267A	Expression strain of AlpH H267A	This study
BL21/pGro7/ pET28a-*alpH* H270A	Expression strain of AlpH H270A	This study
BL21/pGro7/ pET28a-*alpH* D271A	Expression strain of AlpH D271A	This study
BL21/pGro7/ pET28a-*alpH* M174W	Expression strain of AlpH M174W	This study
BL21/pGro7/pET28a-*alpH* D271A/H267A	Expression strain of AlpH D271A/H267A	This study
BL21/pGro7/ pET28a-*alpH* H270A/D271A	Expression strain of AlpH H270A/D271A	This study
BAP1	Used as the host for heterologous expression	[4]
Bjk	BAP1 containing pGro7, pXY-3, pXY-6, pXY-2JK	This study
Bjkh	BAP1 containing pGro7, pXY-3, pXY-6H, pXY-2JK	This study
S. albus J1074	Used as the host for heterologous expression	[5]
WT	BAC plasmid pLXY001 integrated into the chromosome of *S. albus* J1074	[6]
Δ*alp2F-2G*	BAC plasmid pLXY007 integrated into the chromosome of *S. albus* J1074	This study
Δ*alpH*	BAC plasmid pLXY009 integrated into the chromosome of *S. albus* J1074	This study
Δ*alpH::alpH*	Plasmid pPM927-*alpH* integrated into Δ*alpH* mutant	This study
Δ*alp1W*	BAC plasmid pZYC001 integrated into the chromosome of *S. albus* J1074	This study
Δ*alp1W::alp1W*	Plasmid pPM927-*alp1W* integrated into Δ*alp1W* mutant	This study
S. lividans SBT5	Expression strain of Alp1W	[7]
**Plasmids**		
pGro7	GroES- GroEL *ori* Cm^r	TAKARA
pET28a	T7 *lac*, pBR322 origin, Kan^r	Novagen
pET32a	T7 *lac*, pBR322 origin, Amp^r	Novagen
pET28a-*alpH*	pET-28a(+) carrying *alpH*	This study
pET28a-*alpH* K266D	pET-28a(+) carrying *alpH* K266D	This study
pET28a-*alpH* D199A	pET-28a(+) carrying *alpH* D199A	This study

pET28a-*alpH* G203A	pET-28a(+) carrying *alpH* G203A	This study
pET28a-*alpH* H267A	pET-28a(+) carrying *alpH* H267A	This study
pET28a-*alpH* K266A	pET-28a(+) carrying *alpH* K266A	This study
pET28a-*alpH* H270A	pET-28a(+) carrying *alpH* H270A	This study
pET28a-*alpH* D271A	pET-28a(+) carrying *alpH* D271A	This study
pET28a-*alpH* M174W	pET-28a(+) carrying *alpH* M174W	This study
pET28a-*alpH* D271A/H267A	pET-28a(+) carrying *alpH* D271A/H267A	This study
pET28a-*alpH* H270A/D271A	pET-28a(+) carrying *alpH* H270A/D271A	This study
pET28a-*alpJ*	pET-28a(+) carrying *alpJ*	This study
pET28a-*alpK*	pET-28a(+) carrying *alpK*	This study
pXY-2JK	pET28a-*alpAB-MCAT-alpJ-alpK*	This study
pXY-3	pCDFduet-*alpI-ravC*	[8]
pXY-6	pET32a-*alpD-alpE-alpF-alpG*	[8]
pXY-6H	pET32a-*alpD-alpE-alpF-alpG-alpH*	This study
pLXY001	BAC(3C2Δ*alpW*) containing the kinamycin biosynthetic gene cluster	[6]
pLXY007	3C2Δ*alpW*Δ*alp2F-2G*	This study
pLXY009	3C2Δ*alpW*Δ*alpH*	This study
pZYC001	3C2Δ*alpW*Δ*alp1W*	This study
pWY45	o*riT, acc(3)IV, tsr*, for recombinant overexpression	[9]
pWY45-1W	pWY45 carrying *alp1W*	This study
pPM927	Cloning vector, Str^r	[10]
pPM927-*alpH*	*alpH* in pPM927 under the control of *kasO*p*	This study
pPM927-*alp1W*	*alp1W* in pPM927 under the control of its own promoter	This study

Supplementary Table 2. Primers used in this study.

Primer	Sequence (5' to 3')	Description
Δ*alp1W*-F	ACGACAGTGAACTGGAACATCTGGGGCGACG TGGCGGTGATTCCGGGGATCCGTCGACC	Primers for *alp1W* inactivation
Δ*alp1W*-R	CGCCTCGAACAGCGCGACGGTGTCCTCGCCCA GTTCACCTGTAGGCTGGAGCTGCTTC	
Δ*alpH*-F	GAATTGCCGCCTCCGCACGTCGTACG GGAAGCGGAGAAAATTCCGGGGATC CGTCGACC	Primers for *alpH* inactivation
Δ*alpH*-R	GGGGACGGCCTCCACGAGCGAGATC GCCGCCGCCGTCTCTGTAGGCTGGAG CTGCTTC	
alpH-flank-F	TCACACGGGGACGGCCTCC	Primers for Δ*alpH* construct verification
alpH-flank-R	CGAATTGCCGCCTCCGCA	
AlpJ-*Nde*I-F	AAACATATGCCCATCATCTCCGCCGAGGACA	Primers for AlpJ protein expression
AlpJ-*Hind*III-R	AAAAAGCTTCACTCGCCGGCAGAGCGCG	
AlpK-*Nde*I-F	AAACATATGGAATTCTACGATTCAGA	Primers for AlpK protein expression
AlpK-*Hind*III-R	AAAAAGCTTCAGGCGGTGGGGCCGAACC	
AlpH G203A-F	TGGACTTCTGCGGCGCCCAGGGCGCGTTGCT	Primers for AlpH G203A protein expression
AlpH G203A-R	AGCAACGCGCCCTGGGCGCCGCAGAAGTCCA	
AlpH H267A-F	ACGTCCTCAAGGCCATCGTGCA	Primers for AlpH H267A protein expression
AlpH H267A-R	TCGTGCACGATGGCCTTGAGGA	
AlpH K266A-F	CGACGCCTACGTCCTCGCGCACATC	Primers for AlpH K266A protein expression
AlpH K266A-R	AGTCGTGCACGATGTGCGCGAGGAC	
AlpH K266D-F	CGACGCCTACGTCCTCGACCACATC	Primers for AlpH K266D protein expression
AlpH K266D-R	AGTCGTGCACGATGTGGTCGAGGAC	
AlpH H270A-F	TCCTCAAGCACATCGTGGCCGACTG	Primers for AlpH H270A protein expression
AlpH H270A-R	CTCCTCGGGCCAGTCGGCCACGAT	
AlpH D271A-F	CGTGCACGCCTGGCCCGA	Primers for AlpH D271A protein expression
AlpH D271A-R	CGGGCCAGGCGTGCACGA	
AlpH M174W-F	CTTCCAGGGCTGGGGCAGCA	Primers for AlpH M174Wprotein expression
AlpH M174W-R	AGGAGCATCAGGCACAGGTCG	
AlpH H270A/D271A -F	TCCTCAAGCACATCGTGGCCGCCTGGCCCGAG GAGC	Primers for AlpH H270A/D271 protein expression
AlpH H270A/D271A -R	GCTCCTCGGGCCAGGCGGCCACGATGTGCTTG AGGA	
AlpH-*Nde*I-F	AACATATGCCCGAATTGCCGCC	Primers for AlpH protein expression and Δ*alpH* complementation
AlpH-*EcoR*I-R	AGAATTCTCACACGGGGACGGCC	
Alp1W-*Nde*I-F	CCATATGACGCGACCCGTAGT	Primers for Alp1W protein expression
Alp1W-*Hind*III-R	GGAATTCTCAGCGGCGCGGTCC	
Alp1W-onestep -F	CTTTCGTCTTCAAGAATTGGTGAGCCGCAAGG GCACCGGCAGCGGGCCA	Primers for Δ*alp1W* complementation
Alp1W-onestep -R	CGGATGGCGGTGACGAATTCTCAGCGGCGCGG TCCGGCCTTC	

Supplementary Table 3. Protein sequence homology of AlpH homologues.

ID	Identity	Similarity	biosynthesis pathways

TQJ92845.1	67%	78%	uncharacterized
TWD12421.1	67%	78%	uncharacterized
RPE26939.1	72%	85%	uncharacterized
TDB97369.1	56%	71%	nenestatin
SLNWT-5154	60%	74%	fluostatin
Nes22	56%	72%	nenestatin
FlsM	57%	70%	fluostatin
Lom23	55%	69%	lomaiviticin

Supplementary Table 4. Methyltransferases used for phylogenetic analysis and sequence alignment.

methyltransferase	Protein ID
AlpH	8H3T*
Lom23	AHZ61857.1
FlsM	ALJ99866.1
SLNWT-5154	AJE85530.1
IOMT	6CIG*
COMT	1VID*
CHOMT	1FPQ*
SynOMT	3CBG*
LiOMT	2HNK*
SAMT	1M6E*
CCoAOMT	1SUI*
HIOMT	1ZHF*
IAMT	3B5I*
LpCaOMT	3P9C*
DXMT	2EFJ*
Nes22	ARD70871.1
PFOMT	3C3Y*
PdxI	7BQL*
HpiI	7BQO*
ModxI	RYO88689.1
UpiI	XP_002583536.1
EpiI	KAH8701274.1
AdxI	KND87168.1
TQJ92845.1	TQJ92845.1
TWD12421.1	TWD12421.1
RPE26939.1	RPE26939.1
TDB97369.1	TDB97369.1
NNMT	3ROD*
MmcR	3GWZ*
LaPhzM	6C5B*
TPMT	3BGD*

* represents the PDB ID. The others are NCBI accession numbers.

Supplementary Table 5. Statistics of X-ray crystallographic data collection and model refinement.

**Data collection**	
Wavelength (Å)	0.97918
Space group	$P2_12_12_1$
Unit cell parameters	
a, b, c (Å)	68.21, 94.81, 110.11
α, β, γ (°)	90.00, 90.00, 90.00
Resolution range (Å)	71.85 – 1.87 (1.97 – 1.87)
No. of total reflections	749138
No. of unique reflections	59235
I/σ	17.6 (2.7)
Completeness (%)	98.6 (96.7)
R_{merge} (%)[a]	11.0 (95.7)
Redundancy	12.6 (9.9)
**Structure refinement**	
Resolution (Å)	47.73 – 1.87
R_{cryst} / R_{free} (%)[b]	16.22 (28.61)/20.10 (35.60)
RMSD bonds (Å)/angles (°)	0.007/0.870
No. of reflection working set /test set	58926/2998
B-factor (Å^2)	
average	28.76
macromolecules	27.31
ligands	69.35
water	39.41
No. of atoms	
macromolecules	5460
ligands	6
water	719
Ramachandran plot (%)	
most favored	99.16
additionally allowed	0.84
outliers	0.00

[a] $R_{merge} = \sum|I_i - I_m|/\sum I_i$, where I_i is the intensity of the measured reflection and I_m is the mean intensity of all symmetry related reflections.

[b] $R_{cryst} = \Sigma||F_{obs}| - |F_{calc}||/\Sigma|F_{obs}|$, where F_{obs} and F_{calc} are observed and calculated structure factors.

$R_{free} = \Sigma_T||F_{obs}| - |F_{calc}||/\Sigma_T|F_{obs}|$, where T is a test data set of about 5% of the total reflections randomly chosen and set aside prior to refinement.

Numbers in parentheses represent the value for the highest resolution shell.

Supplementary Table 6. ^{1}H NMR and ^{13}C NMR data for glutamylhydrazine in D_2O.

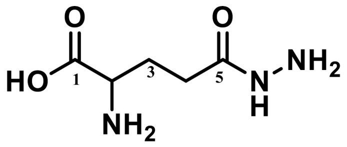

NMR data of glutamylhydrazine

No.	^{1}H, 500 MHz, D_2O	δ_C(^{13}C, 125 MHz, D_2O)
1		173.5
2	3.75 (t, J = 6.2 Hz, 1H)	54.0
3	2.41 – 2.31 (m, 2H)	26.2
4	2.12 (m, 2H)	29.5
5		173.8

Supplementary Table 7. ^{1}H NMR data comparison between reported prekinamycin and our isolated prekinamycin.

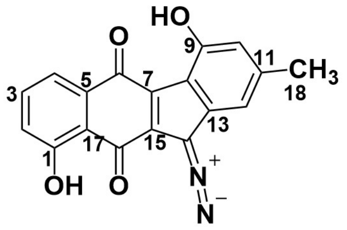

No	This work<br>^{1}H (mult., J in Hz)	Vladimir B. Birman et al. Report,^{1}H
1-OH	12.13 (s,1H)	12.13(s,1H)
9-OH	11.04 (s, 1H)	11.04(s,1H)
3	7.79 (d, J = 7.4 Hz, 1H)	7.79 (d, J = 7.2 Hz, 1H)
2	7.61 (tJ = 7.9 Hz, 1H)	7.60 (t, J = 8.0 Hz, 1H)
4	7.24(overlapped with the $CHCl_3$ peak)	7.24(overlapped with the $CHCl_3$ peak)
10	6.84 (s, 1H)	6.83 (s, 1H)
12	6.71 (s, 1H)	6.71 (s, 1H)
18	2.43 (s, 3H)	2.43 (s, 3H)

Supplementary Table 8. ^{13}C NMR data comparison between reported prekinamycin and our purified prekinamycin.

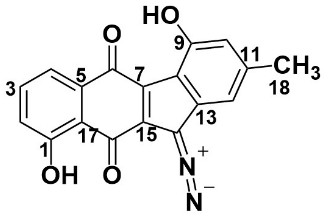

No	This work ^{13}C	Vladimir B. Birman et al. Report, ^{13}C
16	184.8	184.9
6	181.3	181.4
1	162.4	162.5
9	154.7	154.8
11	142.0	142.0
7	136.5	136.5
3	135.9	136.0
15	133.7	133.8
5	131.8	131.9
13	128.4	128.5
2	125.3	125.4
4	120.9	121.0
8	117.7	117.8
17	117.2	115.9
10	115.8	115.5
12	114.2	114.3
14	110.4	110.5
18	29.9	29.9

Supplementary Figures

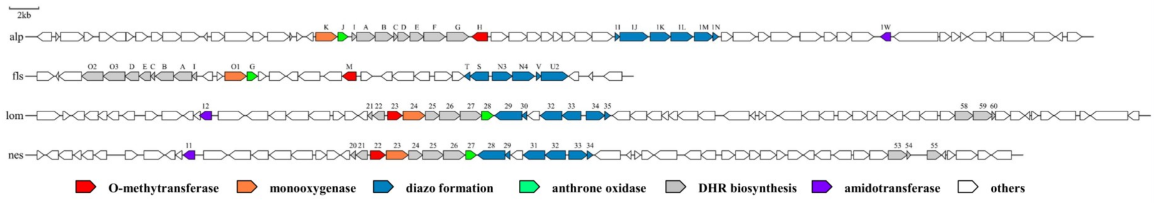

Supplementary Figure 1. The organization of biosynthetic gene clusters. kinamycin (*alp*), fluostatin (*fls*), lomaiviticin (*lom*), and nenestatin (*nes*).

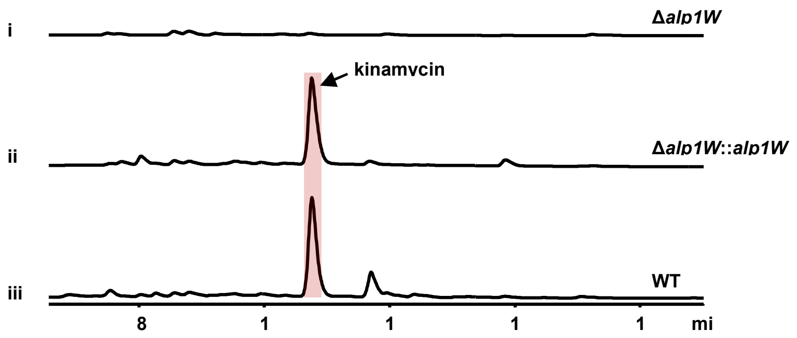

Supplementary Figure 2. ***In vivo*** **characterization of Alp1W**.

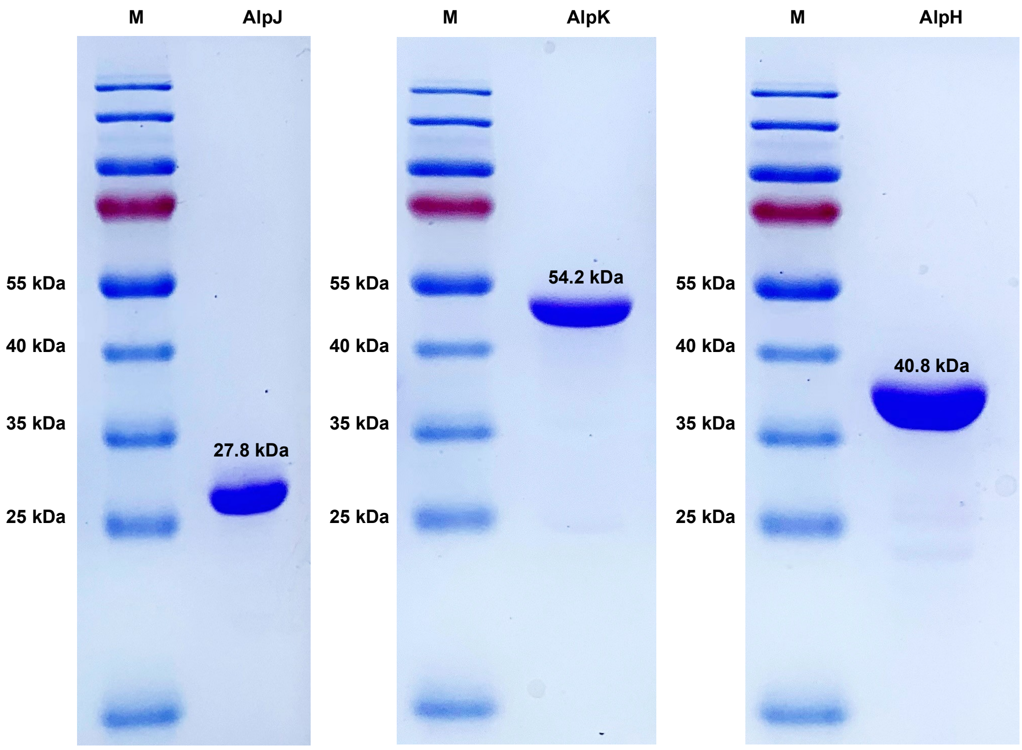

Supplementary Figure 3. SDS-PAGE analysis of purified AlpJ, AlpK, and AlpH. The SDS-PAGE experiments were repeated tree times independently with similar results and the original photographs were supplied at the end of this file. Source data are provided as a Source Data file.

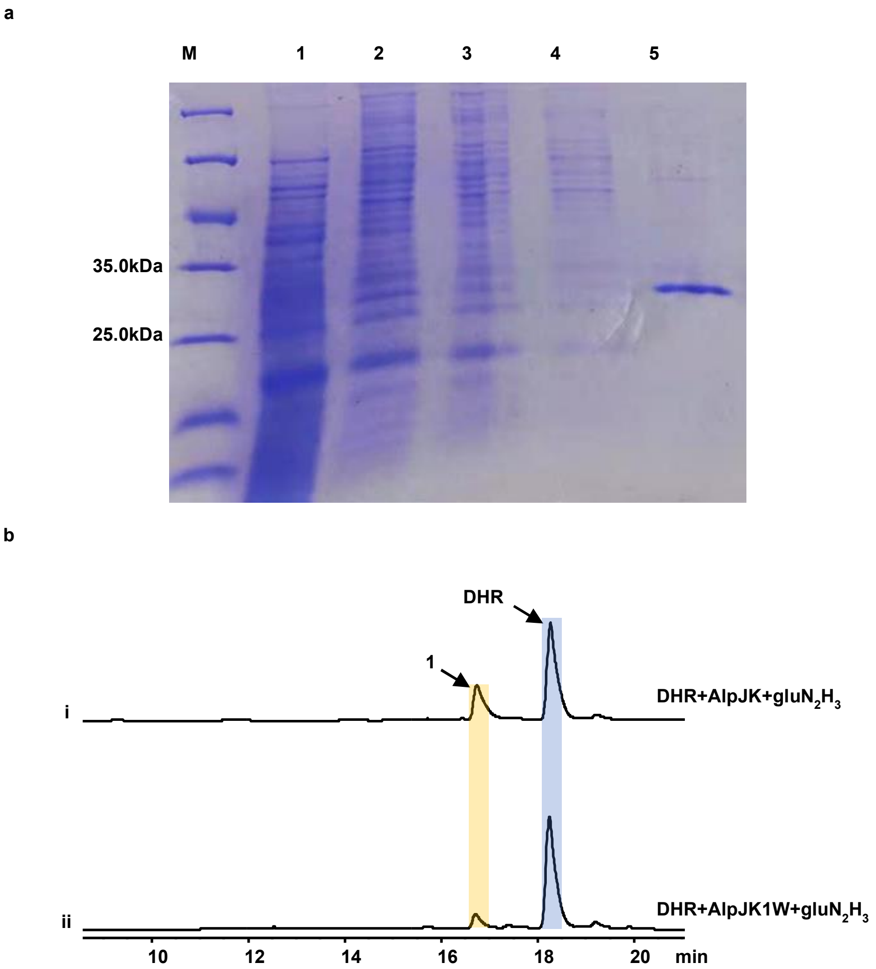

Supplementary Figure 4. ***In vitro*** **characterization of Alp1W.** (a) Purification of Alp1W: Lane1, whole cells; lane 2, supernatant; lane 3, flow through; lane 4, 30mM imidazole elution; lane 5, 500 mM imidazole elution. Source data are provided as a Source Data file. (b) HPLC analysis of the organic extracts of the enzymatic reactions.

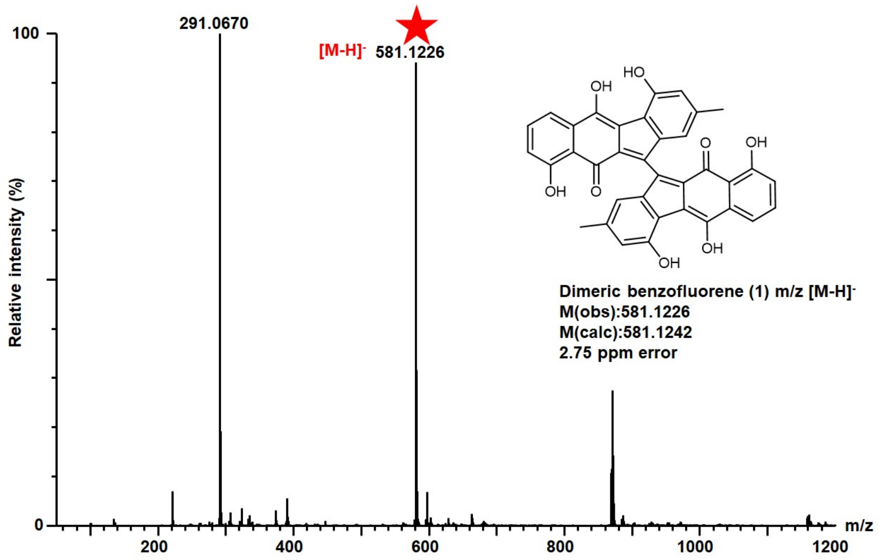

Supplementary Figure 5. LC-HRMS analysis (negative mode) of 1.

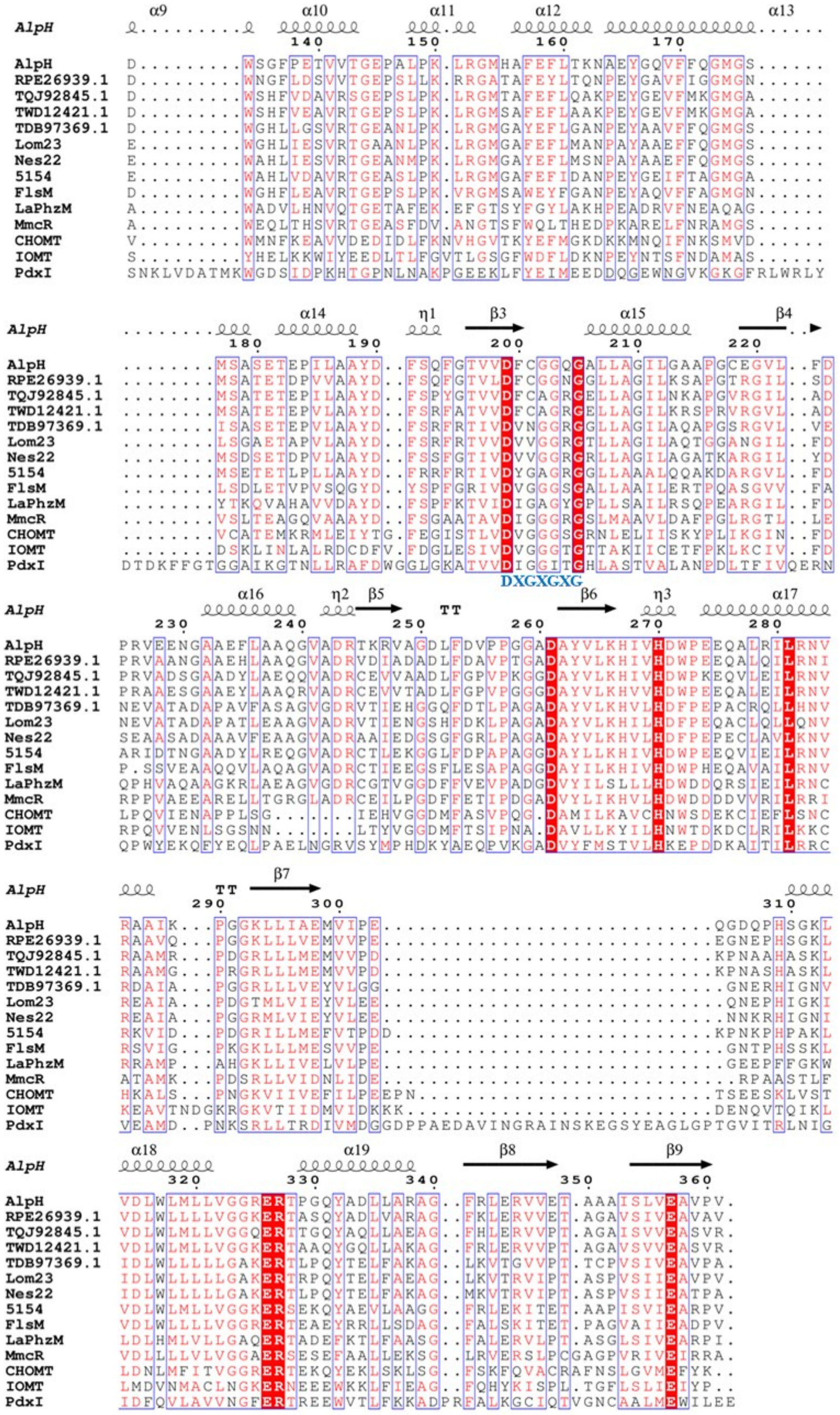

Supplementary Figure 6. Sequence alignment of AlpH and its homologous proteins.

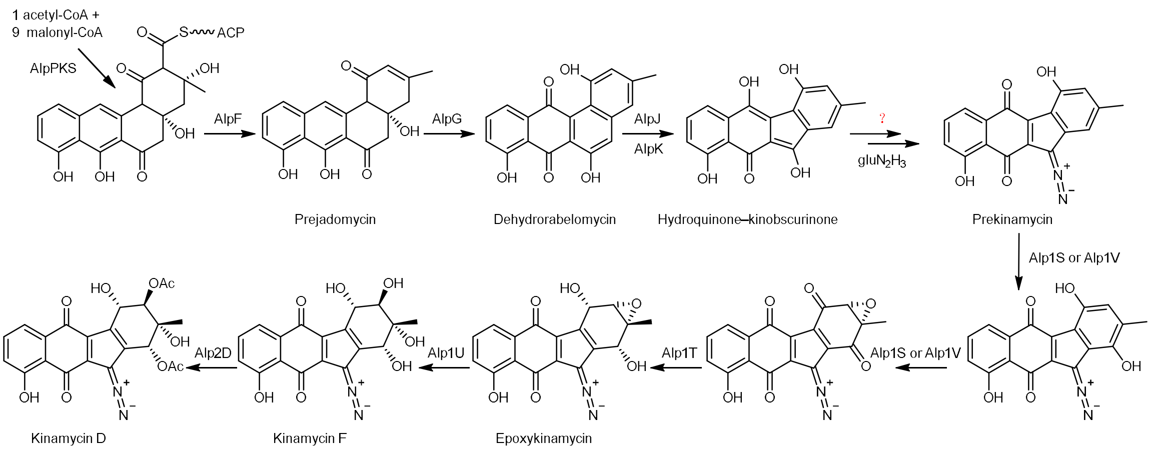

Supplementary Figure 7. Proposed biosynthetic pathway of kinamycin.

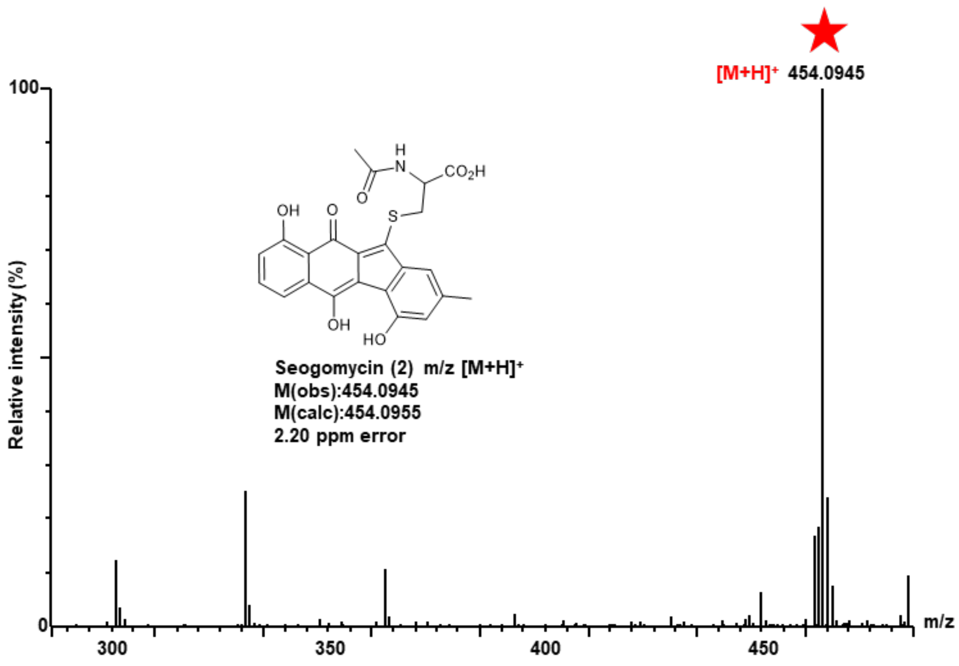

Supplementary Figure 8. HRMS analysis (positive mode) of 2.

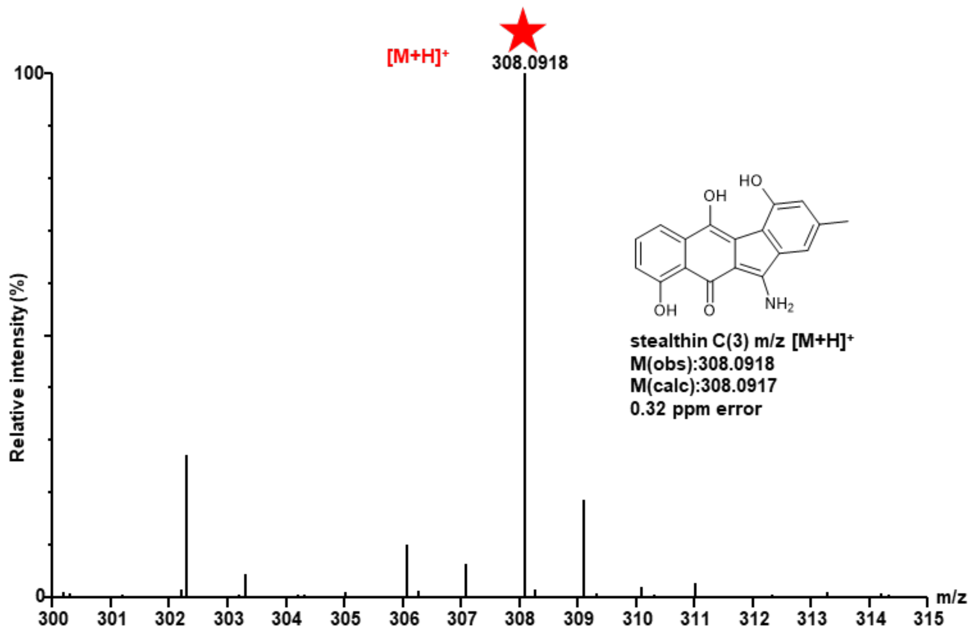

Supplementary Figure 9. HRMS analysis (positive mode) of 3.

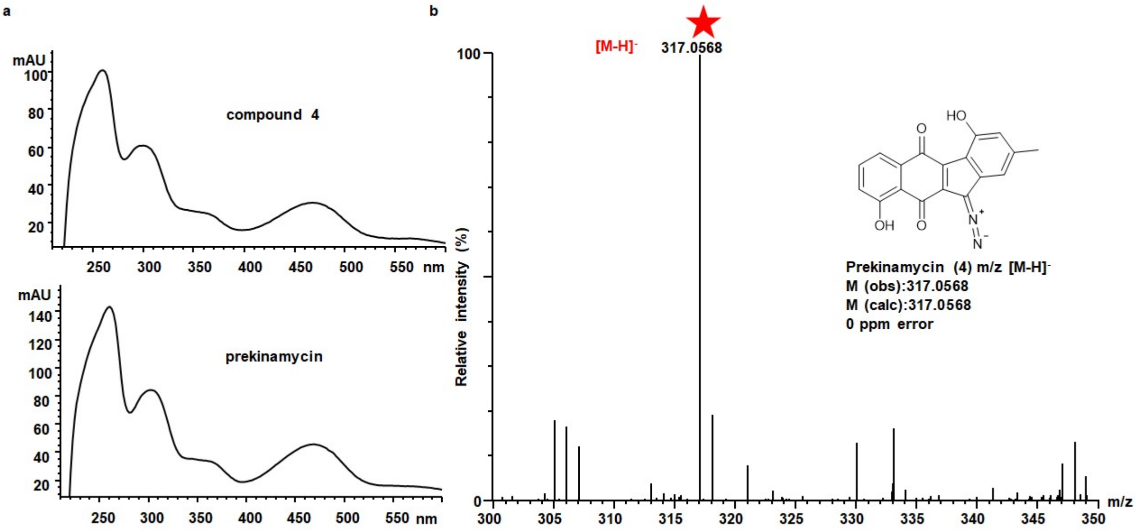

Supplementary Figure 10. UV absorbance spectrum and LC-HRMS analysis of prekinamycin (4). (a) UV absorbance spectrum of **4** and prekinamycin; (b) LC-HRMS data (negative mode) of **4**.

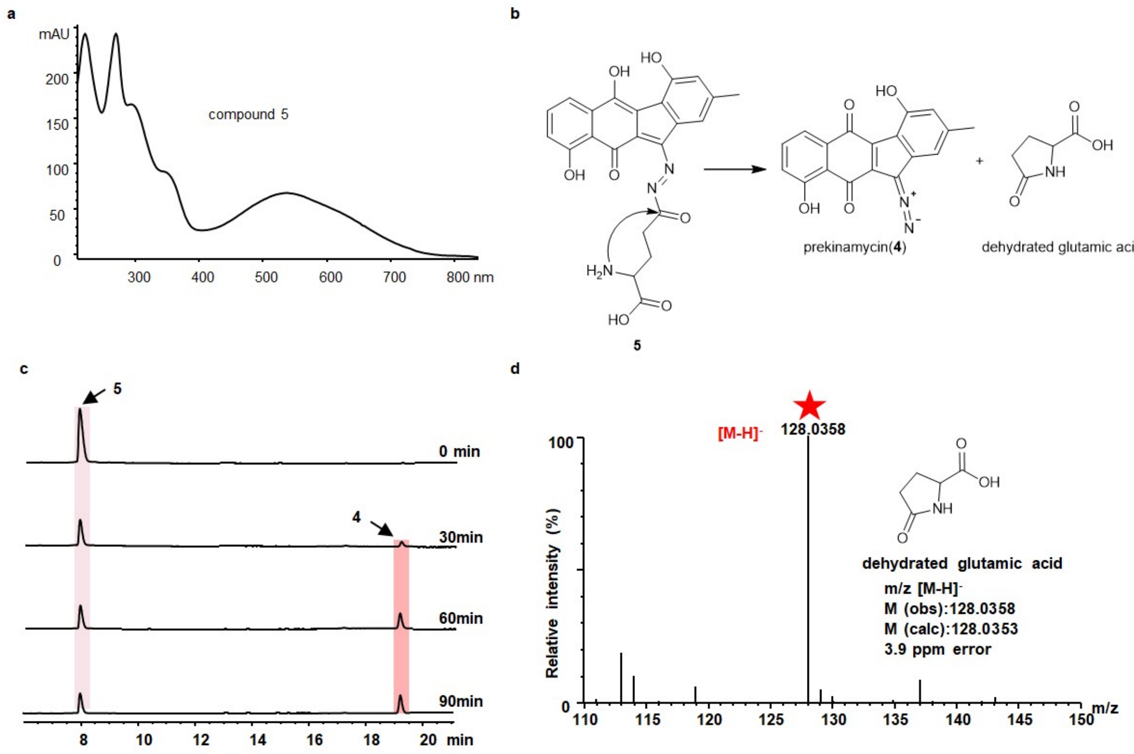

Supplementary Figure 11. HPLC and HRMS analysis of the decomposition products of 5. (a) UV absorbance spectrum of **5**; (b) the decomposition products of **5** and its proposed structure; (c) HPLC analysis of the decomposition products of **5**; (d) MS analysis of the decomposition products of **5**.

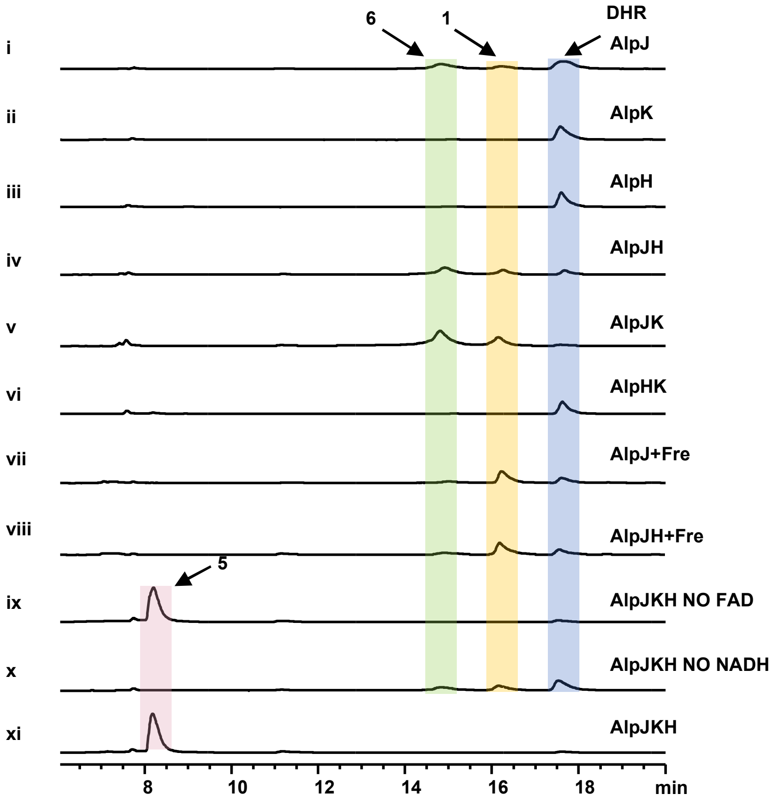

Supplementary Figure 12. HPLC analysis of the reactions with different combinations of enzymes and cofactors for the one-pot reaction. As AlpJ and Fre could catalyze the formation of 6 from DHR, Fre was used to replace AlpK to test the intermediacy of 6.

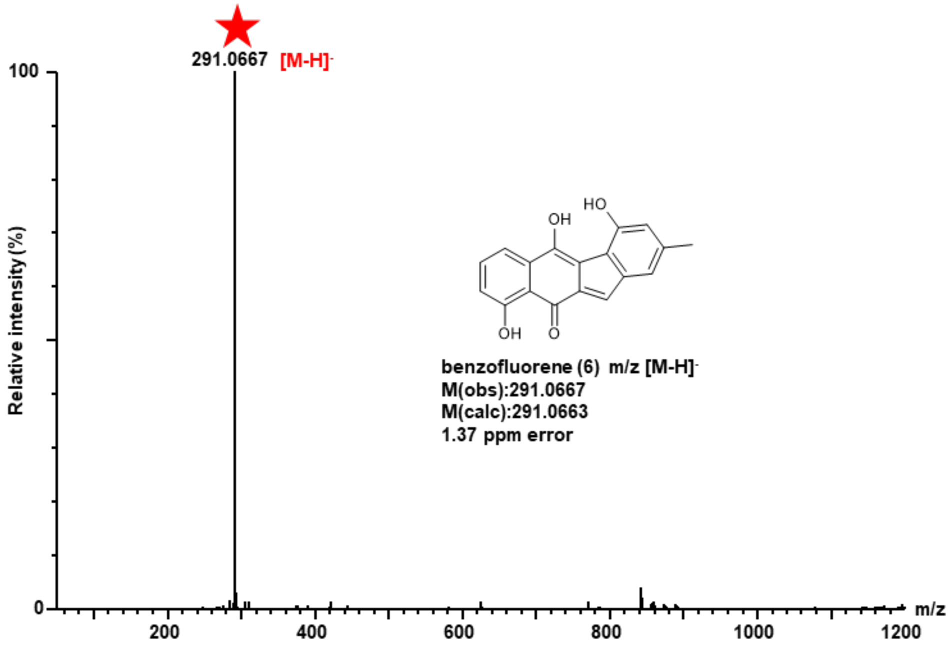

Supplementary Figure 13. LC-HRMS analysis (negative mode) of 6.

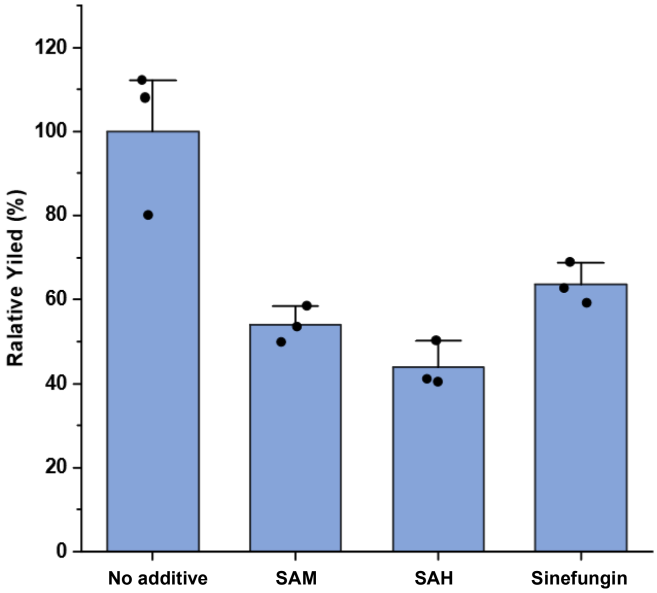

Supplementary Figure 14. Effects of exogenous SAM, SAH, and Sinefungin supplemented to AlpJKH-catalyzed reaction. Reaction conditions:0.25 mM $gluN_2H_3$, 1 mM TCEP, 2 mM NADH, 5 μM FAD, 40 μM AlpJ, 25 μM AlpK, 35 μM AlpH, 120 μM DHR and 1mM SAM/SAH/Sinefungin were incubated in pH 7.5 50 mM Tris-HCl buffer at 30 ℃ for 35 min. The columns represent average values of products (the highest mean value was set 100%); bars indicate ± SD (standard deviation) of n=3 independent replicates. Source data are provided as a Source Data file.

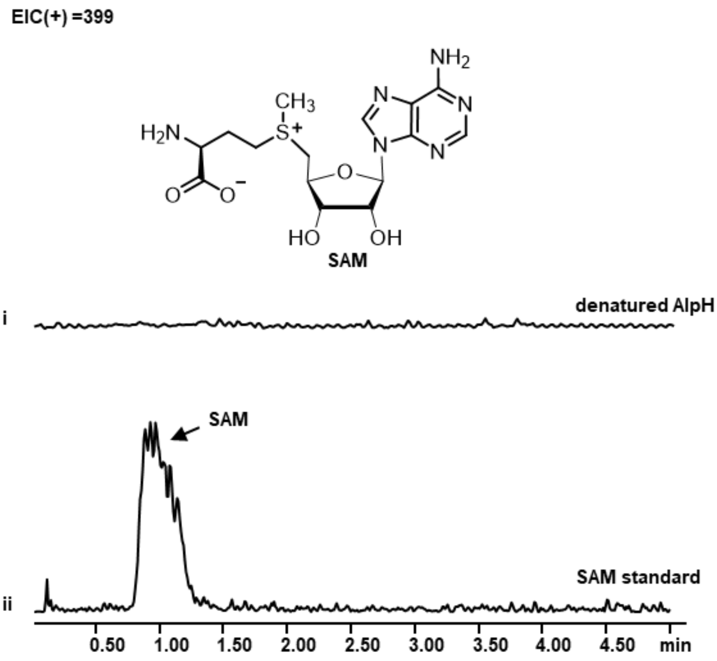

Supplementary Figure 15. LC-MS analysis of chemically denatured AlpH.

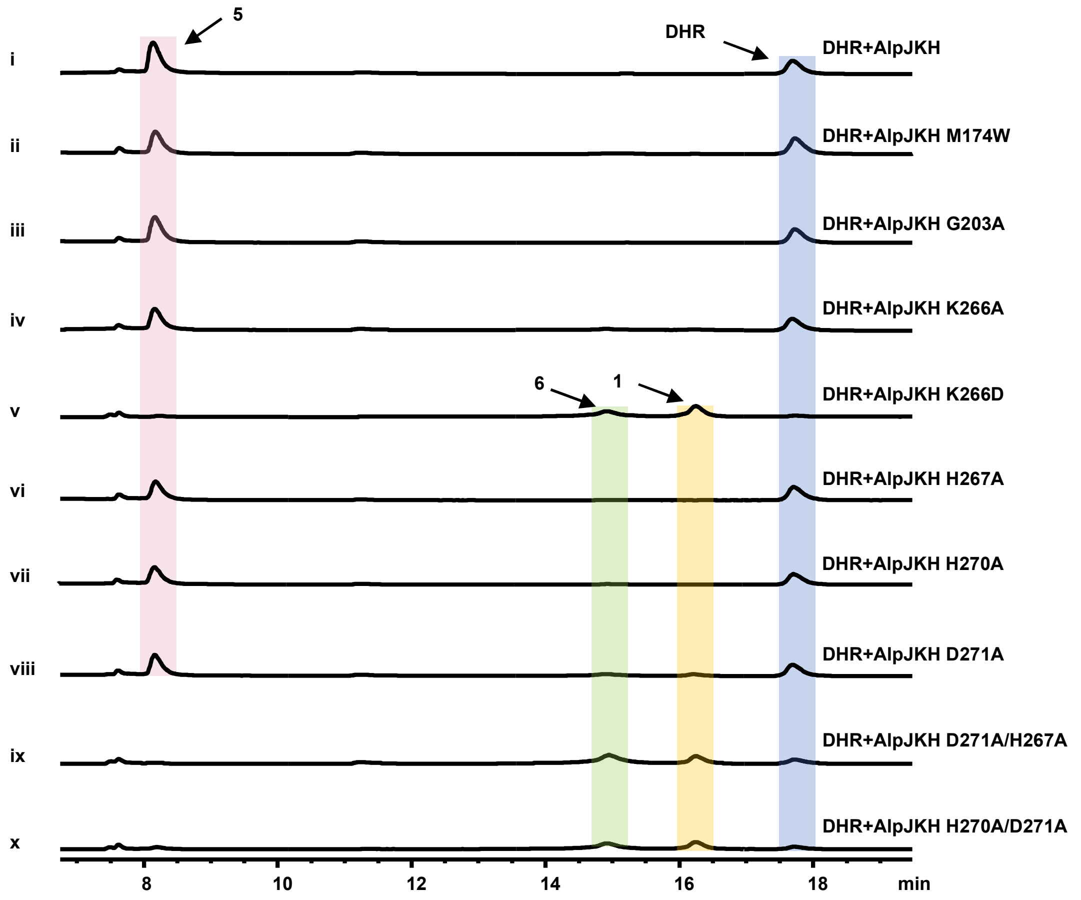

Supplementary Figure 16. HPLC profiles of the reactions catalyzed by wild-type AlpH and its mutants.

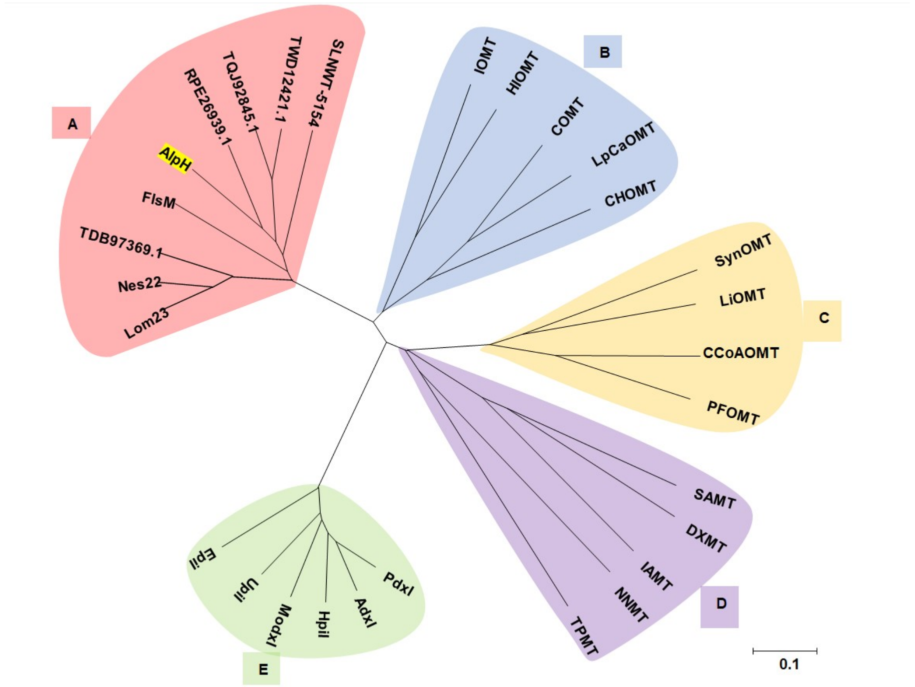

Supplementary Figure 17. Phylogenetic tree of AlpH and its homologues with other natural product methyltransferases (NPMTs). (A) AlpH and its homologous OMT family proteins; (B) OMTs with general acid/base-mediated catalysis mechanism; (C) OMTs with metal-dependent mechanism; (D) NPMTs with proximity and desolvation mechanism; (E) SAM-independent OMT-like enzymes.

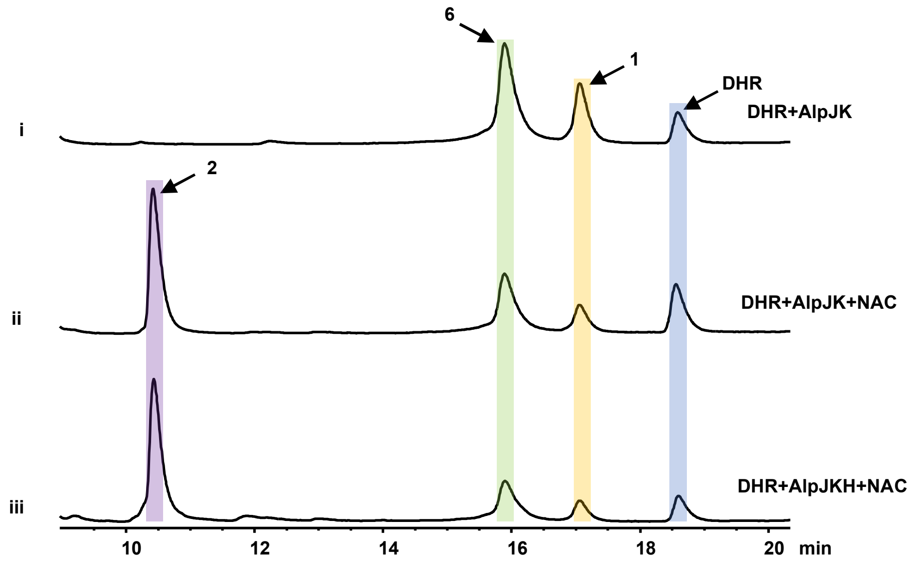

Supplementary Figure 18. AlpH does not affect the production of compound 2. HPLC profiles of DHR + AlpJ + AlpK + NADH (i), DHR + AlpJ + AlpK + NAC (ii), and DHR + AlpJ + AlpK + AlpH + NAC (iii).

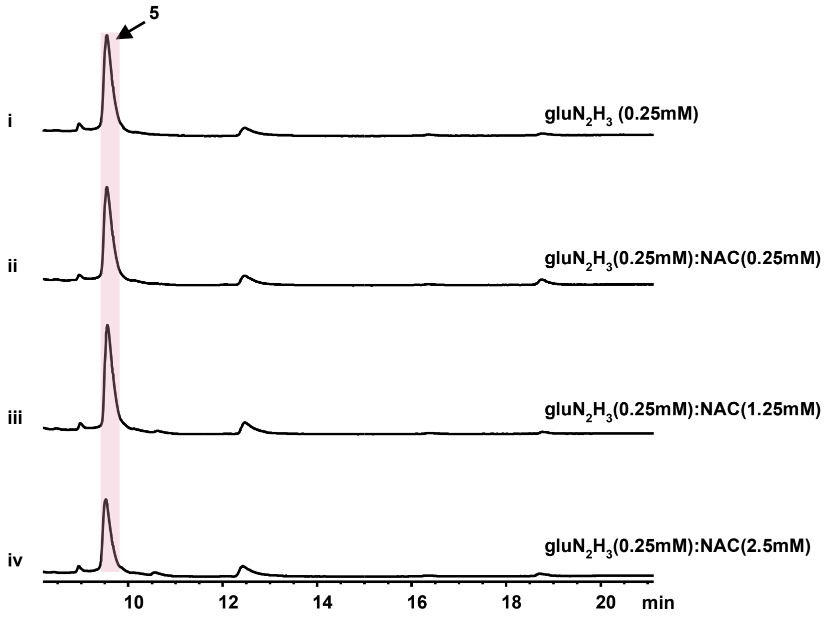

Supplementary Figure 19. HPLC analysis of the competition reactions using different concentrations of NAC. DHR + AlpJ + AlpK + AlpH + NADH + $gluN_2H_3$ with 0 (i), 0.25 **(**ii), 1.25 (iii), and 2.5 (iv) mM NAC.

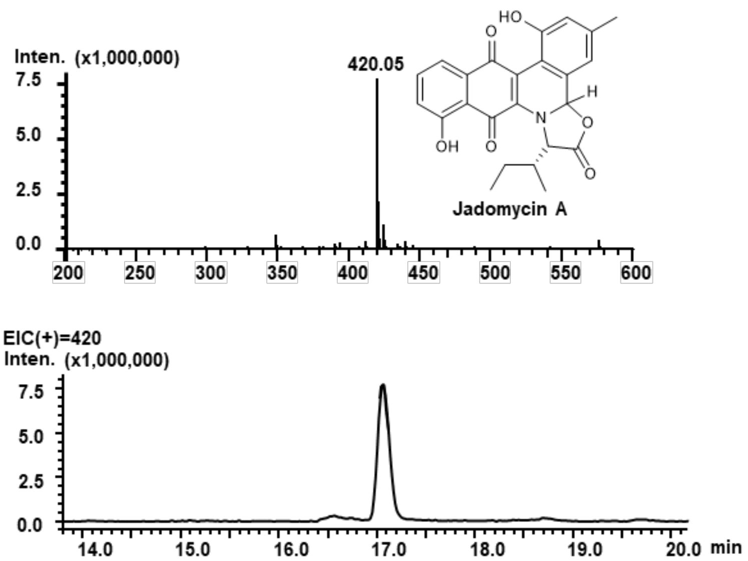

Supplementary Figure 20. Analysis of the effect of isoleucine. LC-MS analysis of DHR + AlpJ + AlpK + Ile reaction.

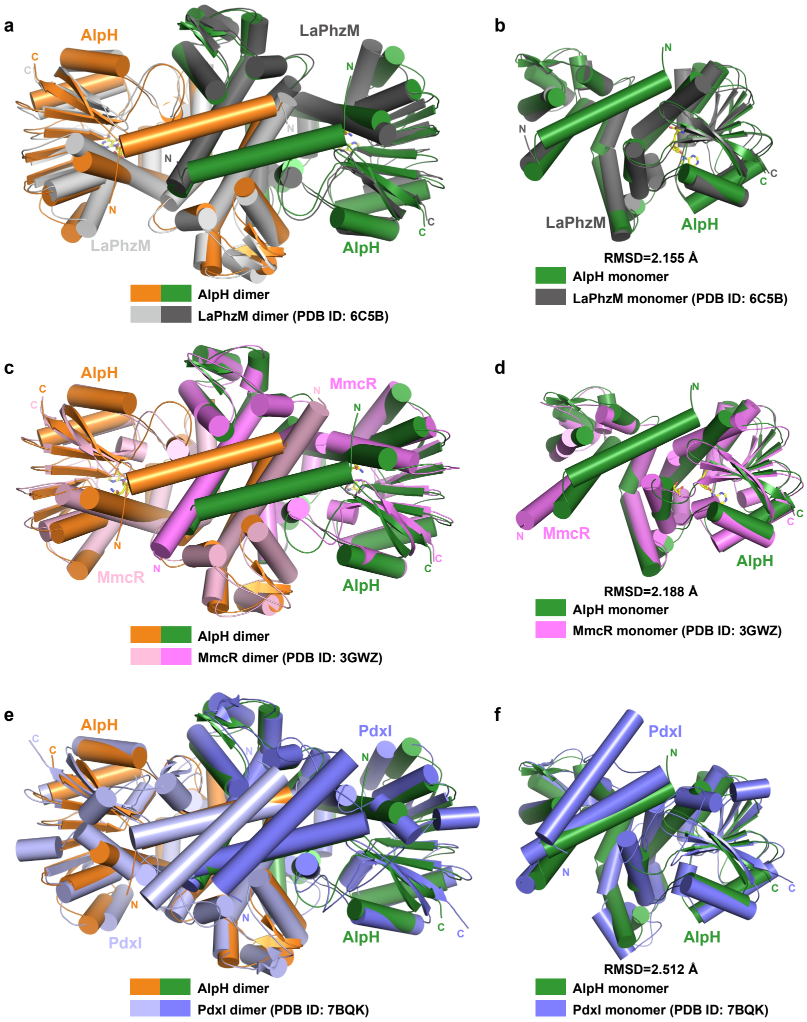

Supplementary Figure 21. Structural comparison analyses of the overall structure of AlpH with the OMT LaPhzM and MmcR as well as the pericyclase PdxI. (a) Ribbon representation showing the overall structural comparison of AlpH and the SAM-dependent OMT LaPhzM in complex with SAH (PDB ID: 6C5B). In this drawing, AlpH is shown in orange and forest green, while LaPhzM is shown in grey and black. (b) Ribbon representation showing the overall structural comparison of the monomeric AlpH and the monomeric LaPhzM in complex with SAH (PDB ID: 6C5B). (c) Ribbon representation showing the overall structural comparison of AlpH and the SAM-dependent OMT MmcR in complex with SAH (PDB ID: 3GWZ). In this drawing, AlpH is shown in orange and forest green, while MmcR is shown in pink and violet. (d) Ribbon representation showing the overall structural comparison of the monomeric AlpH and the monomeric MmcR in complex with SAH (PDB ID: 3GWZ). (e) Ribbon representation showing the overall structural comparison of AlpH and the pericyclase PdxI (PDB ID: 7BQK). In this drawing, AlpH is shown in orange

and forest green, while PdxI is shown in light-blue and slate. (f) Ribbon representation showing the overall structural comparison of the monomeric AlpH and the monomeric PdxI (PDB ID: 7BQK).

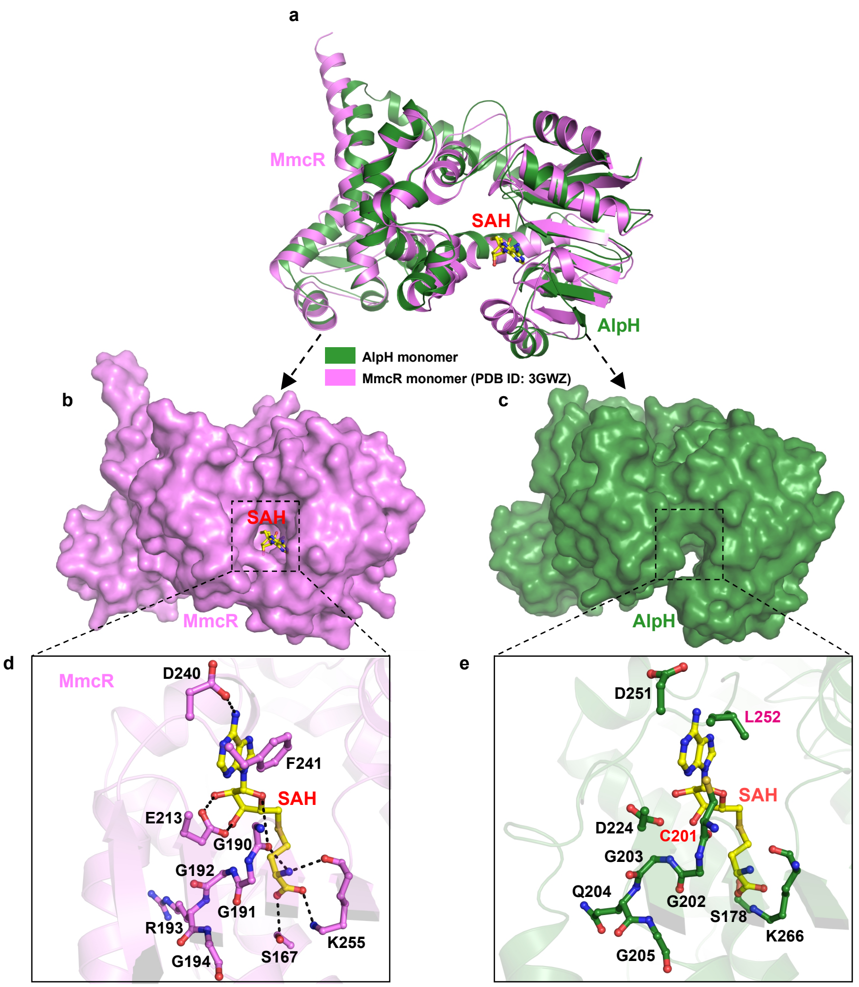

Supplementary Figure 22. Detailed structural comparison of AlpH and the SAM-dependent OMT MmcR in complex with SAH. (a) The combined ribbon diagram and the stick-ball model showing the overall structural comparison of the monomeric AlpH and the monomeric MmcR in complex with SAH (PDB ID: 3GWZ). In this drawing, AlpH is shown in forest green and MmcR in violet, and the bound SAH of MmcR is shown in the stick-ball model. (b) Combination of a surface representation and the stick-ball model showing the SAH-binding pocket of the monomeric MmcR with the same orientation as in panel A. The co-factor SAH of MmcR is shown in the stick-ball model (orange) and is embedded in the pocket located between the N-terminal dimerization domain and the C-terminal catalytic domain of MmcR. (c) The surface representation showing the putative substrate-binding pocket of the monomeric AlpH with the same orientation as in panel A. (d) The combined ribbon diagram and the stick-ball model showing the detailed interactions between the co-factor SAH and MmcR. The critical glycine-rich

(GGGRG) motif of MmcR are also displayed, and the related hydrogen bonds involved in the binding are shown as dotted lines. (E) A structural modeling of AlpH with the co-factor SAH in the MmcR/SAH complex (PDB ID: 3GWZ). The relevant AlpH residues corresponding to the interface residues and the glycine-rich (GGGRG) motif of MmcR for interacting with SAH are shown in the stick-ball model. Notably, the side chain of AlpH Cys201 has a potential steric clash with the SAH molecule.

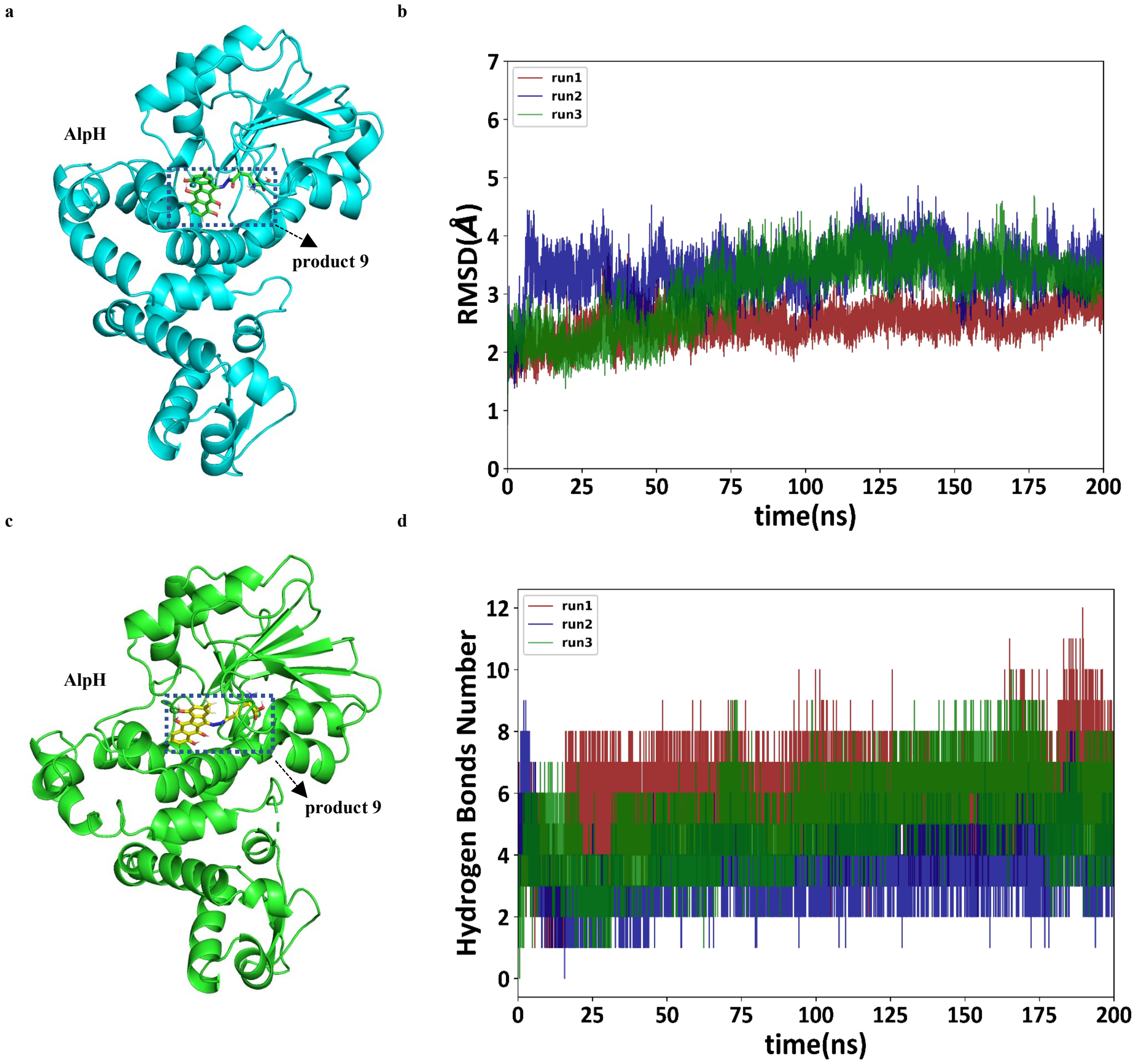

Supplementary Figure 23. MD simulations of the ligand-bound systems. (a) The structure of the ligand-bound state at the beginning of the MD simulation. (b) Time variation of the RMSD of the ligand molecule after superimposing the protein structures along the simulation trajectories. (c) The structure of the ligand-bound state at the end of one MD simulation trajectory. (d) The evolutions of hydrogen bond numbers related to the ligand molecule during the simulations.

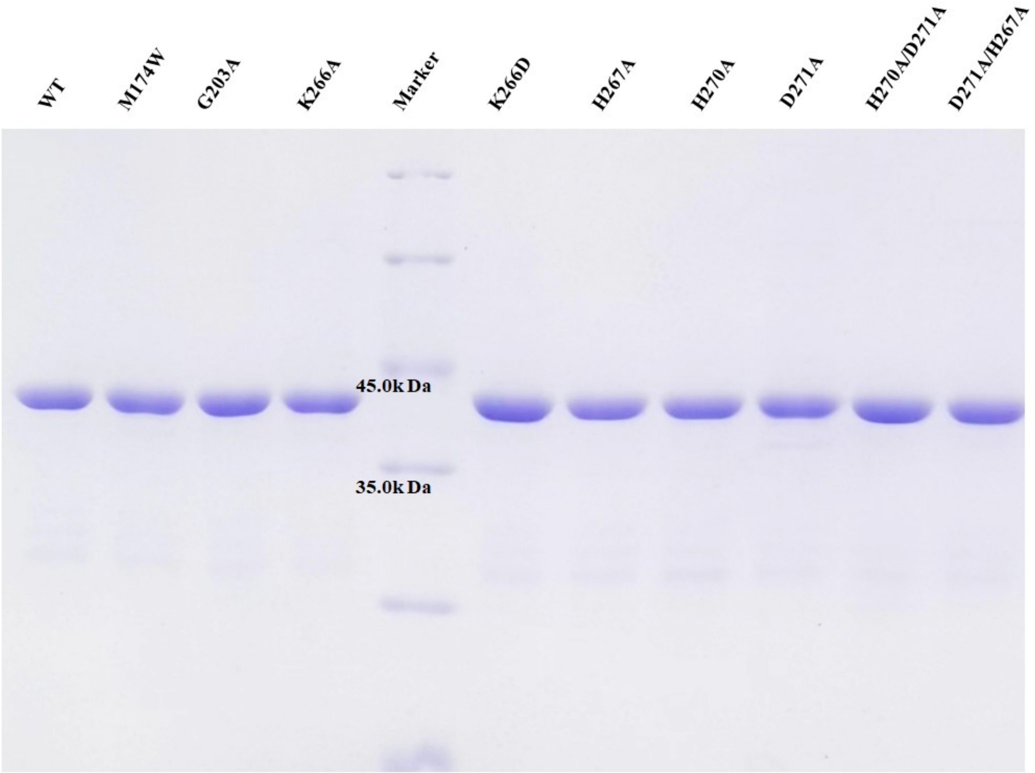

Supplementary Figure 24. SDS-PAGE analysis of purified AlpH and its mutants. The SDS-PAGE experiments were repeated tree times independently with similar results and the original photographs were supplied at the end of this file. Source data are provided as a Source Data file.

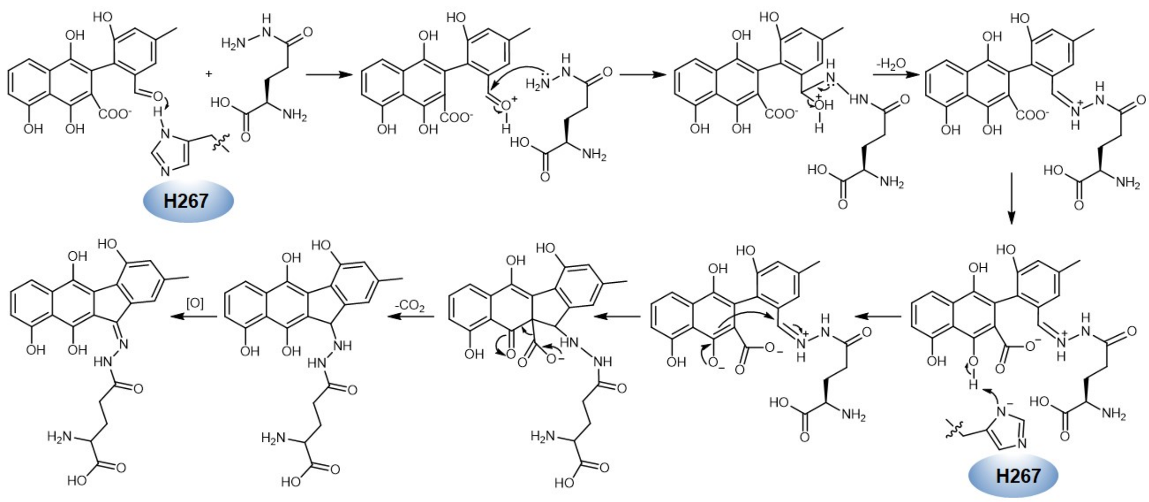

Supplementary Figure 25. Proposed mechanism of C-N and C-C bond formation catalyzed by AlpH.

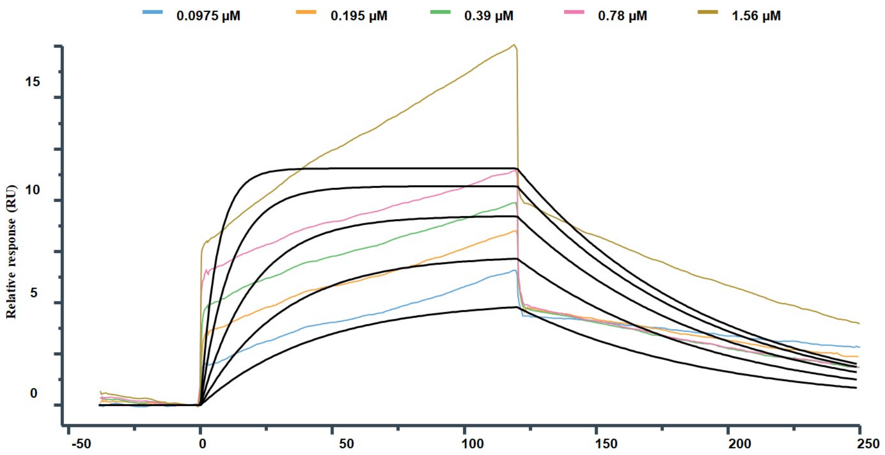

Supplementary Figure 26. Surface plasmon resonance measurement of binding affinity between AlpH and gluN$_2$H$_3$. Representative traces of experiments repeated at least three times showing binding kinetics of different concentrations of gluN$_2$H$_3$ (colored lines) along with best fits using a 1:1 binding model (black lines). K_{on}(1/ms)=1.01×10^5, K_{off}(1/s)=1.15×10^{-2}, K_d(M)=1.14×10^{-7}. Source data are provided as a Source Data file

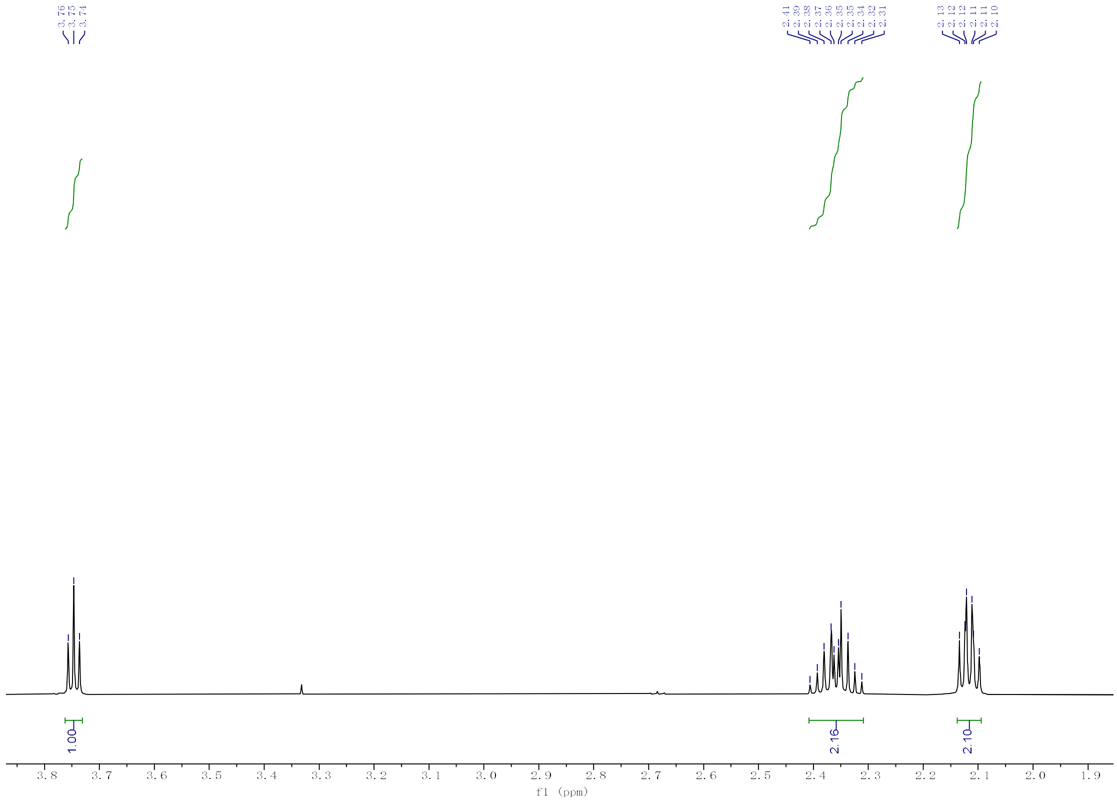

Supplementary Figure 27. ^{1}H NMR spectrum of glutamylhydrazine in D_2O.

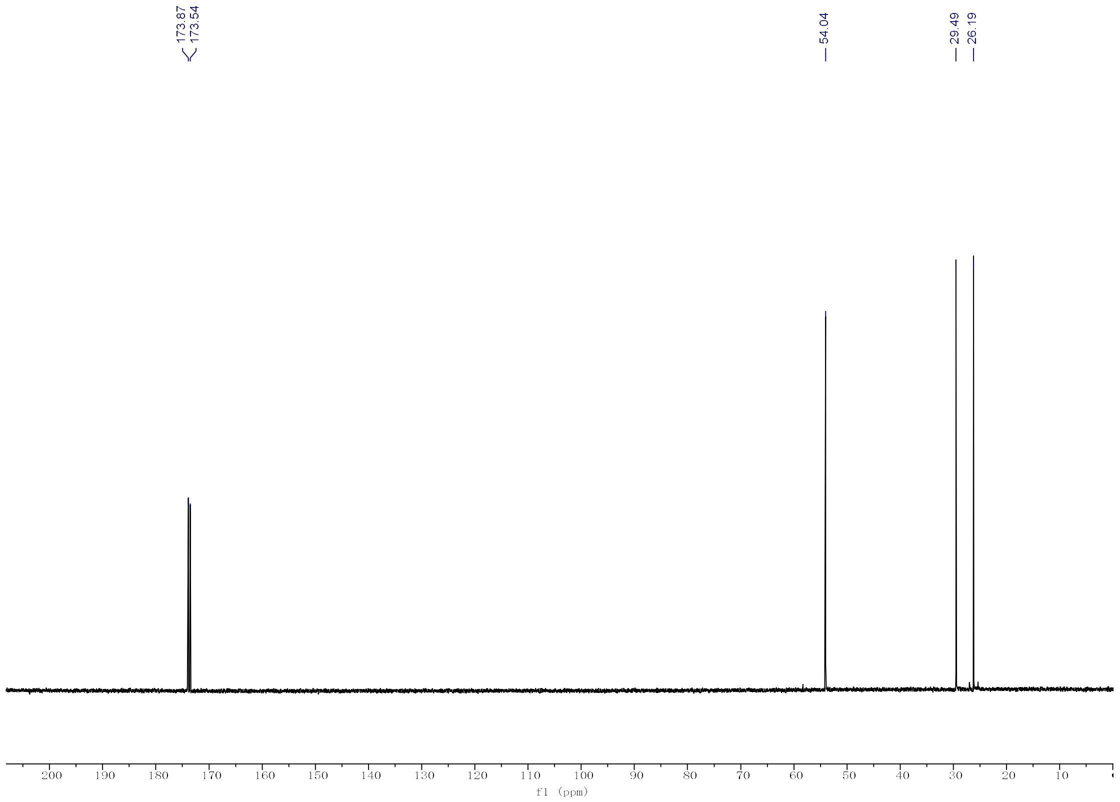

Supplementary Figure 28. ^{13}C NMR spectrum of glutamylhydrazine in D_2O.

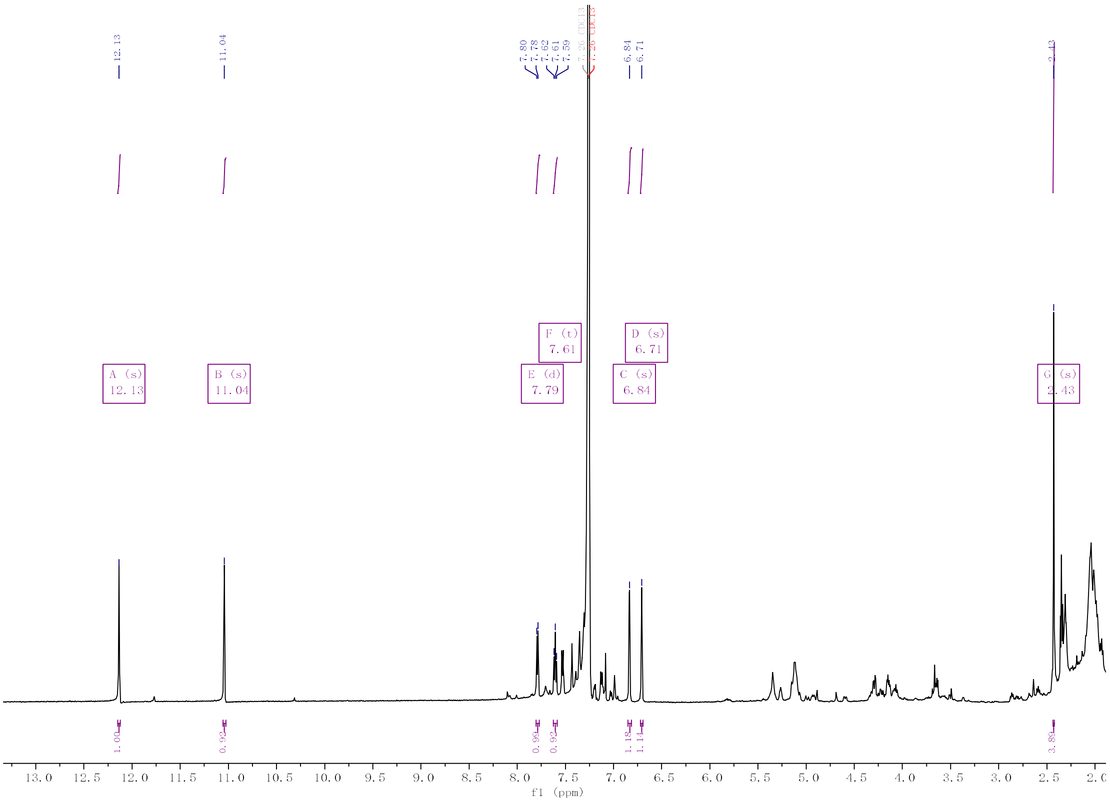

Supplementary Figure 29. ^{1}H NMR spectrum of prekinamycin in $CDCl_3$.

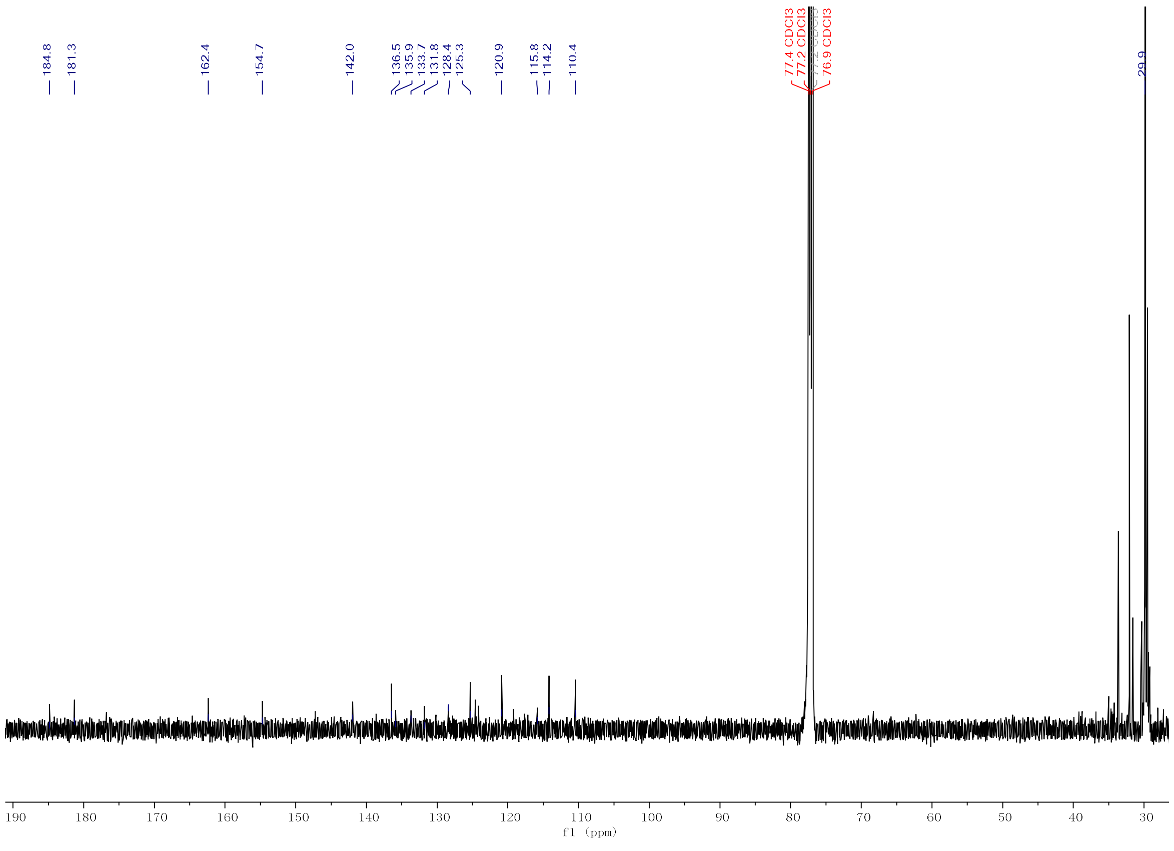

Supplementary Figure 30. ^{13}C NMR spectrum of prekinamycin in $CDCl_3$.

The original SDS-PAGE gels

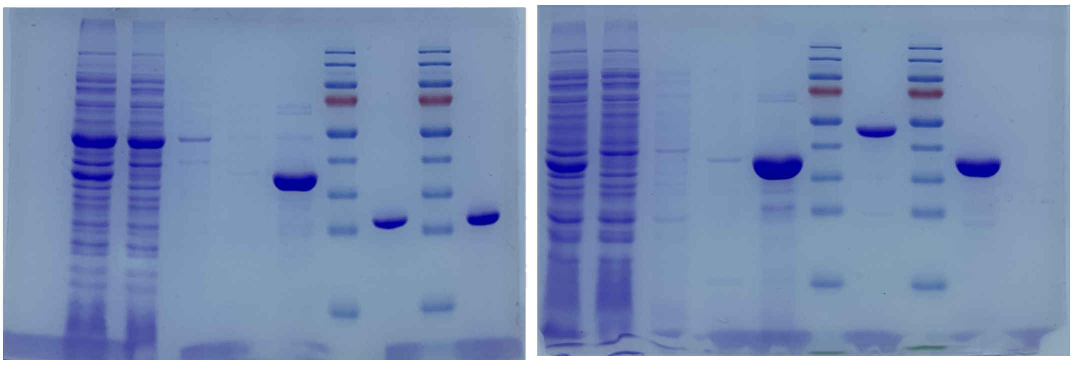

The original SDS-PAGE gels in Supplementary Figure 3

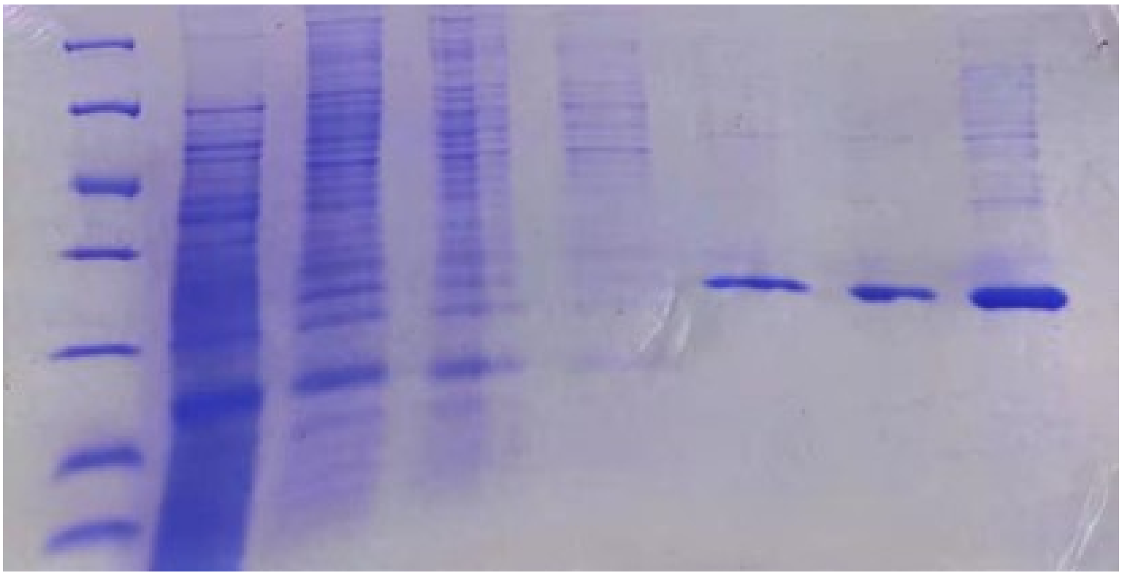

The original SDS-PAGE gels in Supplementary Figure 4

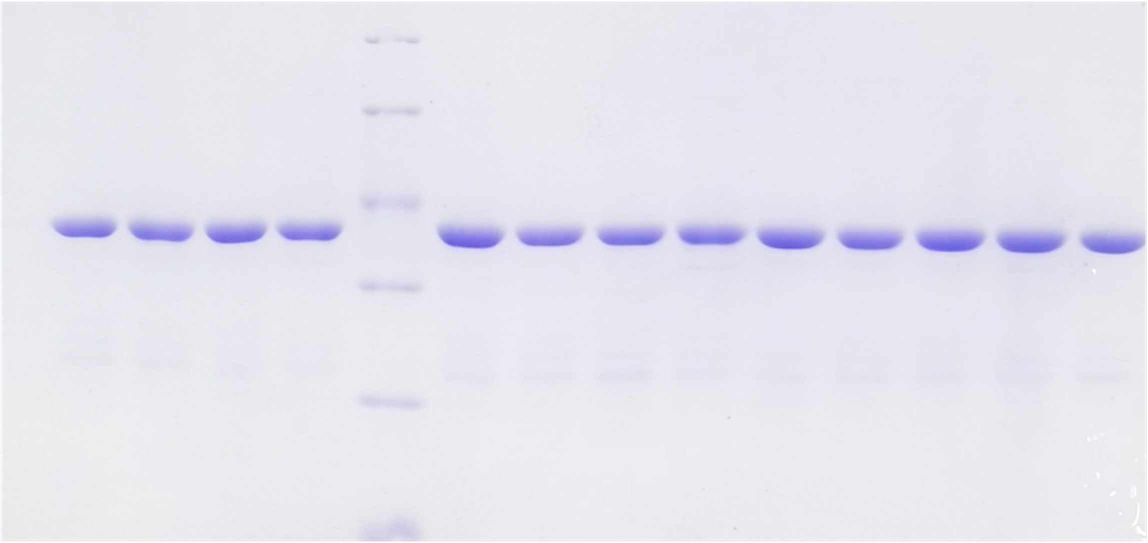

The original SDS-PAGE gels in Supplementary Figure 24

Supplementary references

1 Flett, F., Mersinias, V. & Smith, C. P. High efficiency intergeneric conjugal transfer of plasmid DNA from Escherichia coli to methyl DNA-restricting streptomycetes. *FEMS Microbiol. Lett.* **155**, 223-229, doi:10.1111/j.1574-6968.1997.tb13882.x (1997).

2 Paget, M. S., Chamberlin, L., Atrih, A., Foster, S. J. & Buttner, M. J. Evidence that the extracytoplasmic function sigma factor sigmaE is required for normal cell wall structure in Streptomyces coelicolor A3(2). *J. Bacteriol.* **181**, 204-211, doi:10.1128/jb.181.1.204-211.1999 (1999).

3 Gust, B. *et al.* Lambda red-mediated genetic manipulation of antibiotic-producing Streptomyces. *Adv. Appl. Microbiol.* **54**, 107-128, doi:10.1016/s0065-2164(04)54004-2 (2004).

4 Pfeifer, B. A., Admiraal, S. J., Gramajo, H., Cane, D. E. & Khosla, C. Biosynthesis of complex polyketides in a metabolically engineered strain of E. coli. *Science* **291**, 1790-1792, doi:10.1126/science.1058092 (2001).

5 Chater, K. F. & Wilde, L. C. Streptomyces albus G mutants defective in the SalGI restriction-modification system. *J. Gen. Microbiol.* **116**, 323-334, doi:10.1099/00221287-116-2-323 (1980).

6 Liu, X. *et al.* Reconstitution of Kinamycin Biosynthesis within the Heterologous Host Streptomyces albus J1074. *J. Nat. Prod.* **81**, 72-77, doi:10.1021/acs.jnatprod.7b00652 (2018).

7 Gao, G. *et al.* Formation of an Angular Aromatic Polyketide from a Linear Anthrene Precursor via Oxidative Rearrangement. *Cell Chem Biol* **24**, 881-891.e884, doi:10.1016/j.chembiol.2017.06.008 (2017).

8 Liu, X. *et al.* Heterologous Biosynthesis of Type II Polyketide Products Using E. coli. *ACS Chem. Biol.* **15**, 1177-1183, doi:10.1021/acschembio.9b00827 (2020).

9 Wang, Y. *et al.* Corrigendum: Identifying the Minimal Enzymes for Unusual Carbon-Sulfur Bond Formation in Thienodolin Biosynthesis. *ChemBioChem* **17**, 876, doi:10.1002/cbic.201600175 (2016).

10 Smokvina, T., Mazodier, P., Boccard, F., Thompson, C. J. & Guérineau, M. Construction of a series of pSAM2-based integrative vectors for use in actinomycetes. *Gene* **94**, 53-59, doi:10.1016/0378-1119(90)90467-6 (1990).
