# Supplementary material for: O-methyltransferase-like enzyme catalyzed diazo installation in polyketide biosynthesis
Source: Nat Commun. 2023 Sep 4;14:5372. doi: 10.1038/s41467-023-41062-7 (PMC10477347; doi:10.1038/s41467-023-41062-7)
Supplement: Supplementary file 3 — Reporting Summary [file 41467_2023_41062_MOESM3_ESM.pdf]

Corresponding author(s): Ming Jiang
Last updated by author(s): Jul 31, 2023

Reporting Summary

Nature Portfolio wishes to improve the reproducibility of the work that we publish. This form provides structure for consistency and transparency in reporting. For further information on Nature Portfolio policies, see our Editorial Policies and the Editorial Policy Checklist.

Statistics

For all statistical analyses, confirm that the following items are present in the figure legend, table legend, main text, or Methods section.

n/a	Confirmed	
☐	☒	The exact sample size (*n*) for each experimental group/condition, given as a discrete number and unit of measurement
☐	☒	A statement on whether measurements were taken from distinct samples or whether the same sample was measured repeatedly
☒	☐	The statistical test(s) used AND whether they are one- or two-sided<br>*Only common tests should be described solely by name; describe more complex techniques in the Methods section.*
☒	☐	A description of all covariates tested
☒	☐	A description of any assumptions or corrections, such as tests of normality and adjustment for multiple comparisons
☐	☒	A full description of the statistical parameters including central tendency (e.g. means) or other basic estimates (e.g. regression coefficient) AND variation (e.g. standard deviation) or associated estimates of uncertainty (e.g. confidence intervals)
☒	☐	For null hypothesis testing, the test statistic (e.g. *F*, *t*, *r*) with confidence intervals, effect sizes, degrees of freedom and *P* value noted<br>*Give P values as exact values whenever suitable.*
☒	☐	For Bayesian analysis, information on the choice of priors and Markov chain Monte Carlo settings
☒	☐	For hierarchical and complex designs, identification of the appropriate level for tests and full reporting of outcomes
☒	☐	Estimates of effect sizes (e.g. Cohen's *d*, Pearson's *r*), indicating how they were calculated

Our web collection on statistics for biologists contains articles on many of the points above.

Software and code

Policy information about availability of computer code

Data collection	X-ray data was collected at the beamline BL10U2 of the Shanghai Synchrotron Radiation Facility. The binding affinity was determined by surface plasmon resonance (SPR) using the Biacore 8K. HPLC data were collected by Agilent 1200. LC-MS data was collected by Waters Acquity 2D-UPLC NMR data were collected by Breuker TopSpin 700MHz.
Data analysis	ClustalW(https://www.genome.jp/tools-bin/clustalw), ESPrit 3.0(https://espript.ibcp.fr/ESPript/cgi-bin/ESPript.cgi), Coot 0.8.9, autoPROC 1.0.5, PyMOL 2.5, AutoDock Vina 1.2.0, ChemBio3D Ultra 12.0, BiacoreTM Insight Evaluation Software version 4.0, Gromacs 2018.3, REFMAC 1.17.1, PHENIX1.14, CorelDRAW Graphics Suite 2000, ChemDraw 19.0, MEGA7, MestReNova 11.0.

For manuscripts utilizing custom algorithms or software that are central to the research but not yet described in published literature, software must be made available to editors and reviewers. We strongly encourage code deposition in a community repository (e.g. GitHub). See the Nature Portfolio guidelines for submitting code & software for further information.

Data

Policy information about availability of data

All manuscripts must include a data availability statement. This statement should provide the following information, where applicable:

- Accession codes, unique identifiers, or web links for publicly available datasets
- A description of any restrictions on data availability
- For clinical datasets or third party data, please ensure that the statement adheres to our policy

The coordinate and structure factor of AlpH solved in this study have been deposited in the Protein Data Bank under the accession code PDB ID: 8H3T. All other

relevant data supporting the findings of this study are available in this manuscript and Supplementary Information.

Research involving human participants, their data, or biological material

Policy information about studies with human participants or human data. See also policy information about sex, gender (identity/presentation), and sexual orientation and race, ethnicity and racism.

Reporting on sex and gender	N/A
Reporting on race, ethnicity, or other socially relevant groupings	N/A
Population characteristics	N/A
Recruitment	N/A
Ethics oversight	N/A

Note that full information on the approval of the study protocol must also be provided in the manuscript.

Field-specific reporting

Please select the one below that is the best fit for your research. If you are not sure, read the appropriate sections before making your selection.

☒ Life sciences ☐ Behavioural & social sciences ☐ Ecological, evolutionary & environmental sciences

For a reference copy of the document with all sections, see nature.com/documents/nr-reporting-summary-flat.pdf

Life sciences study design

All studies must disclose on these points even when the disclosure is negative.

Sample size	No statistical method was used to predetermine sample size in this work. Following common practice, the enzyme activities of wild type and each mutants were independently determined in triplicate.
Data exclusions	No data exclusions
Replication	Three independent experiments were conducted, and all attempts at replication were successful. The representative results are displayed in Figure 6e.
Randomization	The experiments were not randomized. In this study, related data resulted from biochemical assays on enzyme function and samples were not allocated to groups.
Blinding	Blinding is not necessary for this research. This is an enzymatic study and appropriate control experiments were included.

Reporting for specific materials, systems and methods

We require information from authors about some types of materials, experimental systems and methods used in many studies. Here, indicate whether each material, system or method listed is relevant to your study. If you are not sure if a list item applies to your research, read the appropriate section before selecting a response.

Materials & experimental systems

n/a	Involved in the study	
☒	☐	Antibodies
☒	☐	Eukaryotic cell lines
☒	☐	Palaeontology and archaeology
☒	☐	Animals and other organisms
☒	☐	Clinical data
☒	☐	Dual use research of concern
☒	☐	Plants

Methods

n/a	Involved in the study	
☒	☐	ChIP-seq
☒	☐	Flow cytometry
☒	☐	MRI-based neuroimaging
